# The prognostic value of preoperative prognostic nutritional index in patients with hypopharyngeal squamous cell carcinoma: a retrospective study

**DOI:** 10.1186/s12967-018-1391-0

**Published:** 2018-01-24

**Authors:** Lu-Lu Ye, Ronald Wihal Oei, Fang-Fang Kong, Cheng-Run Du, Rui-Ping Zhai, Qing-Hai Ji, Chao-Su Hu, Hong-Mei Ying

**Affiliations:** 1 Department of Radiation Oncology, Fudan University Shanghai Cancer Center, 270 Dongan Road, Shanghai, 200032 People’s Republic of China; 2 Department of Head and Neck Surgery, Fudan University Shanghai Cancer Center, 270 Dongan Road, Shanghai, 200032 People’s Republic of China; 3 Department of Oncology, Shanghai Medical College, Fudan University, 270 Dongan Road, Shanghai, 200032 People’s Republic of China

**Keywords:** Prognostic nutritional index, Hypopharyngeal squamous cell carcinoma, Surgery, Prognosis

## Abstract

**Background:**

To analyze the prognostic value of preoperative prognostic nutritional index (PNI) in predicting the survival outcome of hypopharyngeal squamous cell carcinoma (HPSCC) patients receiving radical surgery.

**Methods:**

From March 2006 to August 2016, 123 eligible HPSCC patients were reviewed. The preoperative PNI was calculated as serum albumin (g/dL) × 10 + total lymphocyte count (mm^−3^) × 0.005. These biomarkers were measured within 2 weeks prior to surgery. The impact of preoperative PNI on overall survival (OS), progression-free survival (PFS), locoregional recurrence-free survival (LRFS) and distant metastasis-free survival (DMFS) were analyzed using Kaplan–Meier method and Cox proportional hazards model.

**Results:**

Median value of 52.0 for the PNI was selected as the cutoff point. PNI value was then classified into two groups: high PNI (> 52.0) versus low PNI (≤ 52.0). Multivariate analysis showed that high preoperative PNI was an independent prognostic factor for better OS (P = 0.000), PFS (P = 0.001), LRFS (P = 0.005) and DMFS (P = 0.016).

**Conclusions:**

High PNI predicts superior survival in HPSCC patients treated with radical surgery. As easily accessible biomarkers, preoperative PNI together with the conventional TNM staging system can be utilized to enhance the accuracy in predicting survival and determining therapy strategies in these patients.

## Background

Hypopharyngeal squamous cell carcinoma (HPSCC) is an aggressive tumor with poor prognosis in head and neck squamous cell carcinomas (HNSCC). Since its covert anatomical structures and asymptomatic feature in the early stage, most patients present with advanced stages at primary diagnosis. Moreover, the clinicopathologic characteristics further give rise to poor outcomes on account of extensive submucosal spread, early lymphatic invasion, widely systemic dissemination and high frequency of metachronous or synchronous malignancies [1, 2]. Tumor-node-metastasis (TNM) staging system is by now the main guideline in treatment decision and prognostic prediction for HPSCC patients. Yet, locoregional recurrence and distant failure remain the primary concerns for unfavorable outcomes [2, 3].

Prognostic nutritional index (PNI) is an indicator quantifying the nutritional and immunological status of the body [4]. It was originally designed to evaluate preoperative nutritional conditions and surgical complications in patients with gastrointestinal malignancies [5]. Nowadays, the significance of the PNI as a prognostic predictor has been uncovered in various malignancies [6–8], as well as in HNSCC [9–11]. Nevertheless, its value in HPSCC is scarcely any. We conducted this study to investigate the prognostic value of the PNI in HPSCC patients treated with radical surgery.

## Methods

### Study population

This retrospective study was approved by the Institutional Review Board of Fudan University Shanghai Cancer Center. The study was performed in accordance with the principles of Declaration of Helsinki and its amendments.

Between March 2006 and August 2016, a total of 123 primary HPSCC patients undergoing radical therapy at Fudan University Shanghai Cancer Center were included in this study. The eligibility criteria were: (1) age of 16 years old and above; (2) histologically confirmed squamous cell carcinoma of hypopharyngeal region; (3) completion of the prescribed treatment; (4) complete preoperative blood tests. The exclusion criteria were: (1) presence of distant metastasis or concomitant malignancies at diagnosis; (2) underwent neck nodal dissection prior to surgery; (3) history of head and/or neck irradiation; (4) received preoperative chemotherapy; (5) history of hematological, hepatic or renal diseases; (6) Karnofsky Performance Score < 70.

All patients were screened with full workup before treatment, including: complete medical history, physical examination, electronic laryngoscope, esophageal barium meal examination, contrast-enhanced magnetic resonance imaging (MRI) or computed tomography (CT) scan of the larynx, plain chest CT scan, abdominal sonography, single-photon emission computed tomography (SPECT) of whole body bone scan, as well as hematological tests, including complete blood count, liver and renal function tests. Preoperative total lymphocyte count was measured with automated hematology analyzer Sysmex XT-4000i (Sysmex, Kobe, Japan), while serum albumin level was measured with chemistry analyzer cobas 8000 (Roche, Rotkreuz, Switzerland). The tumor staging was classified using the 7th edition of the American Joint Committee on Cancer (AJCC) staging system for HPSCC.

### Treatment protocol

The treatment modalities for each patient were discussed by our head and neck cancer multidisciplinary team. The team consisted of experienced head and neck surgeons, medical oncologists, radiation oncologists, pathologists and radiologists.

All patients underwent radical pharyngolaryngectomy and cervical lymph node dissection. According to the American Head and Neck Society [12], patients with clinically positive lymph nodes received radical neck dissection, which involves levels I–V. Selective neck dissection was performed in clinically negative lymph node patients, with levels II–IV or II–V involved. Bilateral dissection was carried out in patients with tumors approaching or crossing the midline, or bilateral lymph node metastasis with imaging evidence. Otherwise, unilateral dissection was adopted.

The indications for postoperative radiotherapy were based on the pathological findings, including: (1) residual lesion, (2) primary pathological tumor (pT) 3 or above, (3) close margin (< 5 mm) or positive margin, (4) pathological nodal (pN) 2 or above, (5) extracapsular spread (ECS) of lymph node (LN), (5) perineural invasion, (6) lymphovascular invasion. The radiotherapy was given in the form of intensity-modulated radiotherapy (IMRT) with 6 megavoltage photons. It was performed in a daily fraction of 2.0 Gy, 5 days per week for 6–7 weeks. The prescribed dose was 66–70 Gy to the primary lesion of hypopharynx (GTVnx), 66–70 Gy to the gross tumor volume of metastatic neck lymph nodes (GTVnd), 60 Gy to the high-risk microinvasive areas (clinical target volume 1, CTV1) and 54 Gy to the low-risk areas (clinical target volume 2, CTV2). For patients with positive margin and/or ECS of LN, concurrent chemotherapy with cisplatin was dosed at 80 mg/m^2^ every 3 weeks or 40 mg/m^2^ weekly.

### Follow-up

After the completion of treatment, patients received regular examinations at outpatient clinics at 3-month interval during the first 2 years, every 6–9 months in the 3th–5th years, and annually thereafter. Salvage treatments such as surgery, radiotherapy or systemic chemotherapy were provided to patients with confirmed locoregional relapse or distant metastatic event.

The primary endpoint was overall survival (OS). The secondary endpoints were progression-free survival (PFS), locoregional recurrence-free survival (LRFS) and distant metastasis-free survival (DMFS). OS was defined as the duration from the initiation of treatment to death of any cause. PFS was the time from the beginning of therapy to locoregional relapse or distant metastasis or all-cause death. LRFS was the time interval between the initiation of therapy and the first relapse in hypopharyngeal and/or cervical region. DMFS was the elapsed time between the beginning of treatment and the first occurrence of distant metastasis. For patients who were still alive or with no progressive disease, the latest date of follow up was recorded.

### Statistical analysis

The Statistical Packages for Social Sciences version 22.0 (IBM, Armonk, NY) was used in data analysis. The PNI was dichotomized by its median value. χ^2^ test (or Fischer’s exact test, if indicated) was used to test the baseline balance between high PNI and low PNI subgroups. Survival curves for OS, PFS, LRFS and DMFS were obtained utilizing Kaplan–Meier method. Log-rank test was performed to explore the significance of tested variables on survival outcomes. Univariable and multivariable Cox proportional hazards regression analysis were carried out to assess the significance of variables associated with clinical outcomes. Multivariable analysis included all variables with P value < 0.05 in univariable analysis. Log-minus-log plots was used to evaluate the proportional hazard assumption. Any result with two-sided P value < 0.05 was considered to be statistically significant.

## Results

### Patient characteristics

Baseline characteristics of 123 primary HPSCC patients are listed in Table 1. There were 121 (98.4%) males and 2 (1.6%) females. The median age was 57 years old (range 32–87 years). All patients underwent cervical lymph node dissection, of which 42 (34.1%) received bilateral dissection and 81 (65.9%) patients had unilateral dissection. There were 36 (29.3%) patients with locally advanced diseases (pT3–T4), 103 (83.7%) patients with pathologically confirmed nodal metastasis (pN^+^) of the neck. In terms of TNM staging, there were 5 (4.1%) patients in stage I, 13 (10.5%) in stage II, 20 (16.3%) in stage III and 85 (69.1%) in stage IV. All patients completed the planned course of treatment with 59 (48.0%) patients received radiotherapy alone and 56 (45.5%) patients received combined chemoradiotherapy (CRT) postoperatively.

**Table 1:** Baseline characteristics of 123 patients with hypopharyngeal squamous cell carcinoma

Characteristics	N (%)	PNI		P value^a^
		> 52.0	≤ 52.0	
Age (years)				
≤ 60	87	46	41	
> 60	36	13	23	0.090
Sex				
Male	121 (98.4)	59	62	
Female	2 (1.6)	0	2	0.497
Smoking history				
No	28 (22.8)	13	15	
Yes	95 (77.2)	46	49	0.853
Alcohol history				
No	41 (33.3)	19	22	
Yes	82 (66.7)	40	42	0.799
Pharyngolaryngectomy				
Total	51 (41.5)	28	23	
Partial	72 (58.5)	32	41	0.195
Neck nodal dissection				
Bilateral	42 (34.1)	23	19	
Unilateral	81 (65.9)	36	45	0.277
Tumor differentiation				
Well/moderate	83 (67.5)	38	45	
Poor	40 (32.5)	21	19	0.485
Primary tumor site				
Pyriform sinus	108 (87.8)	53	55	
Posterior wall/postcricoid	15 (12.2)	6	9	0.510
pT classification^b^				
T1–T2	87 (70.7)	43	44	
T3–T4	36 (29.3)	16	20	0.615
pN classification^b^				
N0	20 (16.3)	11	9	
N1–3	103 (83.7)	48	55	0.491
pTNM staging^b^				
I–II	18 (14.6)	10	8	
III–IV	105 (85.4)	49	56	0.486
No. of metastatic LN				
≤ 3	98 (79.7)	46	52	
> 3	25 (20.3)	13	12	0.651
LND				
≤ 0.06	66 (53.7)	31	35	
> 0.06	57 (46.3)	28	29	0.812
ECS of LN				
Negative	104 (84.6)	50	54	
Positive	19 (15.4)	9	10	0.955
Surgical margin				
Negative	115 (93.5)	55	60	
Positive	8 (6.5)	4	4	1.000^b^
Perineural invasion				
Negative	108 (87.8)	52	56	
Positive	15 (12.2)	7	8	0.914
Lymphovascular invasion				
Negative	99 (80.5)	46	53	
Positive	24 (19.5)	13	11	0.498
Adjuvant treatment				
No	8 (6.5)	4	4	
RT alone	59 (48.0)	30	29	
CRT	56 (45.5)	29	31	0.796
Disease progression				
Absence	56 (45.5)	38	18	
Presence	67 (54.5)	21	46	*0.000*

Italic values indicate significance of p value (p < 0.05)

CRT, combined chemoradiotherapy; ECS, extracapsular spread; LN, lymph node; LND, lymph node density; PNI, prognostic nutritional index; pT classification, pathological tumor classification; pN classification, pathological nodal classification; RT, radiotherapy; TNM, tumor-node-metastasis

^a^Chi-square (χ^2^) test, P < 0.05

^b^Tumor-node-metastasis staging system proposed by the American Joint Committee on Cancer (7th edition)

The median value of 52.0 (range 41.6–60.2) was selected as cutoff point for PNI. We classified preoperative PNI into two groups: high PNI (> 52.0) versus low PNI (≤ 52.0). As depicted in Table 1, there were insignificant differences in the distribution of clinicopathological characteristics between the PNI groups, with the exception of disease progression (P = 0.000).

On the whole, during a median follow-up of 39.5 months (range 4.3–142.3 months), 36 (29.3%) patients experienced locoregional recurrence. The mean time to locoregional recurrence was 33.7 months (range 3.7–142.3 months). There were 49 (39.8%) patients developed distant metastasis, of which lung was the most common site of metastasis, followed by bone. Meanwhile, 19 (15.4%) patients had both locoregional and distant metastatic events. Among 19 (15.4%) patients who occurred second primary tumors, there were 14 (73.7%) patients developed tumor arising from upper aerodigestive tract. A total of 56 (45.5%) patients were dead. The 5-year OS, PFS, LRFS and DMFS were 52.9, 47.7, 65.0 and 58.8%, respectively.

### Univariable and multivariable analysis

**Fig. 1:**
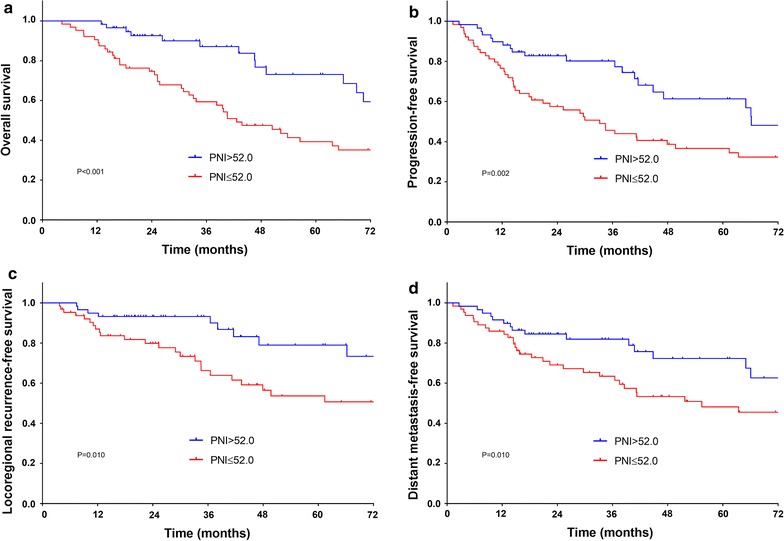
Kaplan–Meier survival curves of overall survival (**a**), progression-free survival (**b**), locoregional recurrence-free survival (**c**) and distant metastasis-free survival (**d**) according to preoperative prognostic nutritional index (PNI). Log-rank test, P < 0.05

Kaplan–Meier method with log-rank test (Fig. 1) showed that compared to patients with low PNI, those with high PNI were significantly superior in 5-year OS (73.1% versus 39.3%, P = 0.000), PFS (61.3% versus 36.6%, P = 0.002), LRFS (79.0% versus 53.7%, P = 0.010) and DMFS (72.3% versus 48.2%, P = 0.010).

Cox univariable analysis (Table 2) revealed that inferior OS was associated with primary tumor site at posterior wall/postcricoid (posterior wall/postcricoid versus pyriform sinus, P = 0.000), positive ECS of LN (positive versus negative, P = 0.007) and surgical margin (positive versus negative, P = 0.001), high lymph node density (LND) (> 0.06 versus ≤ 0.06, P = 0.020) and low PNI (P = 0.001). As to PFS, it was inferior in primary tumor at posterior wall/postcricoid (P = 0.000), advanced pT classification (T1–T2 versus T3–T4, P = 0.009), positive surgical margin (P = 0.032), high LND (0.005) and low PNI (P = 0.003). LRFS was significantly shortened in tumor from posterior wall/postcricoid (P = 0.000), advanced pT (P = 0.000), positive surgical margin (P = 0.011) and low PNI (P = 0.013). With regard to DMFS, advanced age (> 60 versus ≤ 60, P = 0.015), tumor at posterior wall/postcricoid (P = 0.018), positive pN classification (N0 versus N1–N3, P = 0.032) and ECS of LN (P = 0.022), late pathological TNM (pTNM) staging (I–II versus III–IV, P = 0.046), high number of metastatic LN (> 3 versus ≤ 3, P = 0.015), high LND (P = 0.012) and low PNI (P = 0.029) had significant correlation with worse survival.

**Table 2:** Univariate analysis of survival outcomes of 123 patients with hypopharyngeal squamous cell carcinoma

Variables	Overall survival (OS)		Progression-free survival (PFS)		Locoregional recurrence-free survival (LRFFS)		Distant metastasis-free survival (DMFS)	
	HR (95% CI)^a^	P value	HR (95% CI)^a^	P value	HR (95% CI)^a^	P value	HR (95% CI)^a^	P value
Age								
≤ 60	Ref		Ref		Ref		Ref	
> 60	1.538 (0.882–2.681)	0.129	1.427 (0.197–10.338)	0.725	0.782 (0.355–1.723)	0.541	2.035 (1.147–3.611)	*0.015*
Sex								
Male	Ref		Ref		Ref		Ref	
Female	1.980 (0.271–14.445)	0.500	1.467 (0.202–10.632)	0.705	2.782 (0.377–20.549)	0.316	1.963 (0.269–14.322)	0.506
Smoking history								
No	Ref		Ref		Ref		Ref	
Yes	1.480 (0.746–2.934)	0.262	1.460 (0.796–2.677)	0.221	1.385 (0.606–3.166)	0.440	0.910 (0.482–1.716)	0.770
Alcohol history								
No	Ref		Ref		Ref		Ref	
Yes	0.950 (0.546–1.654)	0.857	1.029 (0.617–1.715)	0.914	1.837 (0.837–4.035)	0.130	0.744 (0.418–1.322)	0.313
Tumor differentiation								
Well/moderate	Ref		Ref		Ref		Ref	
Poor	1.143 (0.659–1.981)	0.634	1.131 (0.680–1.881)	0.635	0.940 (0.461–1.913)	0.864	1.376 (0.771–2.456)	0.280
Primary tumor site								
Pyriform sinus	Ref		Ref		Ref		Ref	
Posterior wall/postcricoid	3.652 (1.885–7.077)	*0.000*	3.274 (1.789–5.994)	*0.000*	4.508 (2.075–9.793)	*0.000*	2.451 (1.168–5.141)	*0.018*
pT classification^b^								
T1–T2	Ref		Ref		Ref		Ref	
T3–T4	1.643 (0.963–2.802)	0.068	1.906 (1.171–3.102)	*0.009*	3.258 (1.687–6.294)	*0.000*	1.452 (0.811–2.598)	0.209
pN classification^b^								
N0	Ref		Ref		Ref		Ref	
N1–N3	1.242 (0.561–2.747)	0.593	1.416 (0.676–2.966)	0.357	0.613 (0.279–1.348)	0.224	4.701 (1.141–19.380)	*0.032*
pTNM staging^b^								
I–II	Ref		Ref		Ref		Ref	
III–IV	1.699 (0.678–4.262)	0.258	1.819 (0.785–4.212)	0.163	1.060 (0.412–2.729)	0.904	4.217 (1.023–17.378)	*0.046*
No. of metastatic LN								
≤ 3	Ref		Ref		Ref		Ref	
> 3	1.479 (0.780–2.807)	0.231	1.463 (0.822–2.606)	0.196	1.000 (0.415–2.406)	0.999	2.168 (1.161–4.046)	*0.015*
LND								
≤ 0.06	Ref		Ref		Ref		Ref	
> 0.06	1.886 (1.107–3.214)	*0.020*	2.010 (1.236–3.268)	*0.005*	1.319 (0.686–2.537)	0.406	3.020 (1.661–5.493)	*0.000*
ECS of LN								
Negative	Ref		Ref		Ref		Ref	
Positive	2.222 (1.243–3.971)	*0.007*	1.710 (0.974–3.000)	0.062	1.026 (0.427–2.468)	0.954	2.102 (1.114–3.967)	*0.022*
Surgical margin								
Negative	Ref		Ref		Ref		Ref	
Positive	3.695 (1.656–8.244)	*0.001*	2.374 (1.078–5.228)	*0.032*	3.466 (1.336–8.991)	*0.011*	1.853 (0.662–5.191)	0.240
Perineural invasion								
Negative	Ref		Ref		Ref		Ref	
Positive	1.645 (0.775–3.491)	0.195	1.133 (0.540–2.376)	0.741	0.520 (0.12402.169)	0.369	1.231 (0.523–2.900)	0.634
Lymphovascular invasion								
Negative	Ref		Ref		Ref		Ref	
Positive	1.779 (0.951–3.328)	0.071	1.406 (0.778–2.542)	0.259	0.483 (0.148–1.581)	0.229	1.564 (0.795–3.075)	0.195
Adjuvant treatment								
No	Ref		Ref		Ref		Ref	
RT alone	2.584 (0.620–10.770)	0.192	3.098 (0.745–12.884)	0.120	1.944 (0.461–8.193)	0.365	4.177 (0.567–30.747)	0.160
CRT	2.125 (0.490–9.212)	0.314	3.301 (0.777–14.015)	0.105	0.884 (0.188–4.170)	0.877	4.830 (0.644–36.237)	0.126
PNI								
> 52.0	Ref		Ref		Ref		Ref	
≤ 52.0	2.804 (1.529–5.140)	*0.001*	2.202 (1.313–3.693)	*0.003*	2.526 (1.217–5.241)	*0.013*	2.177 (1.185–4.000)	*0.012*

Italic values indicate significance of p value (p < 0.05)

CI, confidence interval; CRT, combined chemoradiotherapy; ECS, extracapsular spread; HR, hazard ratio; LN, lymph node; LND, lymph node density; PNI, prognostic nutritional index; pT classification, pathological tumor classification; pN classification, pathological nodal classification; Ref, reference; RT, radiotherapy; TNM, tumor-node-metastasis

^a^Cox proportional hazards model. Bolding shows P value < 0.05

^b^Tumor-node-metastasis staging system proposed by the American Joint Committee on Cancer (7th edition)

Multivariable Cox proportional hazards regression analysis further analyzed including variables with P value < 0.05 in univariate analysis (Table 3). Primary tumor at posterior wall/postcricoid (HR 2.590, 95% CI 1.252–5.358; P = 0.010), positive surgical margin (HR 2.842, 95% CI 1.080–7.482; P = 0.034) and low PNI (HR 3.842, 95% CI 1.963–7.518; P = 0.000) were independent prognostic factors for shortened OS. As for PFS, primary tumor at posterior wall/postcricoid (HR 2.328, 95% CI 1.224–4.426; P = 0.010), advanced pT (HR 1.842, 95% CI 1.115–3.042; P = 0.017), high LND (HR 1.971, 95% CI 1.201–3.237; P = 0.007) and low PNI (HR 2.401, 95% CI 1.419–4.061; P = 0.001) remained significantly associated with inferior survival. Primary tumor at posterior wall/postcricoid (HR 2.608, 95% CI 1.163–5.851; P = 0.020), advanced pT (HR 3.063, 95% CI 1.559–6.015; P = 0.001), positive surgical margin (HR 3.455, 95% CI 1.253–9.526; P = 0.017) and low PNI (HR 2.958, 95% CI 1.388–6.307; P = 0.005) were independent risk factors for worsened LRFS. Advanced age (HR 2.510, 95% CI 1.376–4.580; P = 0.003), primary tumor at posterior wall/postcricoid (HR 2.914, 95% CI 1.296–6.553; P = 0.010), high LND (HR 2.430, 95% CI 1.206–4.896; P = 0.013) and low PNI (HR 2.133, 95% CI 1.154–3.943; P = 0.016) were still independently correlated with decreased DMFS.

**Table 3:** Multivariate analysis of survival outcomes of 123 patients with hypopharyngeal squamous cell carcinoma

Variables	Overall survival (OS)		Progression-free survival (PFS)		Locoregional recurrence-free survival (LRFFS)		Distant metastasis-free survival (DMFS)	
	HR (95% CI)^a^	P value	HR (95% CI)^a^	P value	HR (95% CI)^a^	P value	HR (95% CI)^a^	P value
Age								
≤ 60							Ref	
> 60							2.510 (1.376–4.580)	*0.003*
Primary tumor site								
Pyriform sinus	Ref		Ref		Ref		Ref	
Posterior wall/postcricoid	2.590 (1.252–5.358)	*0.010*	2.328 (1.224–4.426)	*0.010*	2.608 (1.163–5.851)	*0.020*	2.914 (1.296–6.553)	*0.010*
pT classification^b^								
T1–T2			Ref		Ref			
T3–T4			1.842 (1.115–3.042)	*0.005*	3.063 (1.559–6.015)	*0.001*		
pN classification^b^								
N0							Ref	
N1–N3							2.519 (0.335–18.963)	0.370
pTNM staging^b^								
I–II							Ref	
III–IV							1.126 (0.151–8.402)	0.908
No. of metastatic LN								
≤ 3							Ref	
> 3							1.497 (0.731–3.066)	0.269
LND								
≤ 0.06	Ref		Ref				Ref	
> 0.06	1.959 (0.959–2.974)	0.070	1.971 (1.201–3.237)	*0.007*			2.430 (1.206–4.896)	*0.013*
ECS of LN								
Negative	Ref						Ref	
Positive	1.670 (0.866–3.222)	0.126					1.326 (0.665–2.647)	0.423
Surgical margin								
Negative	Ref		Ref		Ref			
Positive	2.842 (1.080–7.482)	*0.034*	1.790 (0.767–4.179)	0.178	3.455 (1.253–9.526)	*0.017*		
PNI								
> 52.0	Ref		Ref		Ref		Ref	
≤ 52.0	3.842 (1.963–7.518)	*0.000*	2.401 (1.419–4.061)	*0.001*	2.958 (1.388–6.307)	*0.005*	2.133 (1.154–3.943)	*0.016*

Italic values indicate significance of p value (p < 0.05)

CI, confidence interval; ECS, extracapsular spread; HR, hazard ratio; LN, lymph node; LND, lymph node density; PNI, prognostic nutritional index; pT classification, pathological tumor classification; pN classification, pathological nodal classification; Ref, reference; TNM, tumor-node-metastasis

^a^Cox proportional hazards model. Bolding shows P value < 0.05

^b^Tumor-node-metastasis staging system proposed by the American Joint Committee on Cancer (7th edition)

## Discussion

The treatment modalities for HPSCC patients are mostly based on multidisciplinary approach. Radical pharyngolaryngectomy and cervical lymph node dissection with/without adjuvant radiotherapy or chemoradiotherapy is one of main traditional approaches. The 5-year OS in our study was 52.9%, while previous studies reported that it ranged from 41 to 55% [3, 13–18] with radical surgery as the main therapy modality. Our study presented that the preoperative PNI was an effective factor in predicting outcomes for HPSCC patients with radical surgery, in terms of OS, LRFS, DMFS and PFS. Table 1 indicated that parameters were similar between high PNI and low PNI groups, except disease progression (P = 0.000). No significant association between PNI and conventional prognostic predictors, such as: T classification, N classification, TNM stage and others, was found. Our finding was consistent with previous studies, which were done with a second validation cohort and also showed little or no correlation between PNI and other conventional prognostic predictors [19, 20]. This might be due to different focus of both predictors, where conventional prognostic predictors focus merely on tumor behavior, while PNI indicates both immunonutritional status of the host and reflect the systemic inflammation [4, 21]. Of note, patients with high PNI had better locoregional and distant control. Conversely, 71.9% (46/64) patients in low PNI group developed disease progression. Current therapy protocol guided by TNM staging does not have an effective tumor control in low PNI group.

PNI index was designed by Buzby [5] in 1980. It was initially applied to evaluate surgical complications and mortality in patients with gastrointestinal malignancies. Henceforth, it was widely validated as an independent prognostic indicator for postoperative complications and treatment outcomes in various malignancies [6, 9–11]. The calculation of PNI incorporates serum albumin concentration and total lymphocyte count in peripheral blood. It reflects nutritional and immunological status of the host [4]. As for HNSCC, 25–50% of patients present with initial nutritional deterioration at diagnosis, while a tumor arising from hypopharyngeal region is of particular predominant [22]. As a matter of fact, tumor invasion can bring about stenosis of upper aerodigestive tract which mechanically interferes with normal chewing and swallowing. Treatment-related malnutrition is another concern. Tumor resection will damage normal structure to some degree. Consequent adjuvant therapy such as radiotherapy or chemotherapy will further exacerbate the conditions of dysphagia, odynophagia or anorexia because of change in taste, mucositis, fibrosis, et al. In turn, compromised nutritional status will lead to suboptimal treatment or discontinuation of further therapy. As a result, malnutritional status has been proved profoundly to be correlated with deteriorated quality of life and outcomes in HNSCC patients [23, 24], as well as in HPSCC [25]. Serum albumin is a known indicator reflecting the state of nutrition [26]. Hypoalbuminemia were demonstrated with increased tumor progression and poor survival in cancer patients [27]. Recent prospective study conducted by Kühn [28] further corroborated the impact of hypoalbuminemia on tumor occurrence and mortality.

On the other hand, immune system is of crucial importance in tumor surveillance [29], individuals with immunosuppression [30] or immunodeficiencies [31] have been illustrated with higher risk of cancer development. As the vital components of cellular adaptive immune function, lymphocytes play an indispensable role in immune surveillance to defend tumor cell invasion [32]. Lymphopenia is not only associated with a higher risk of neoplasms [33] and earlier tumor progression [34], but also predicts the poor outcomes in malignancies [35].

Taken together, PNI is a comprehensive index which can give an objective assessment of nutritional and immunological condition. Moreover, malnutrition exerts an undesirable impact on immunity, nutritional support can modulate immune function [36]. To our knowledge, this is by now the first study reporting the prognostic value of preoperative PNI in HPSCC patients with radical surgery. Though Lo WC [15] once reported that neutrophil to lymphocyte ratio (NLR) is a prognostic indicator of survival in HPSCC, several studies illustrated that PNI is more superior compared to other inflammatory and nutritional indexes in predicting survival, including NLR, platelet to lymphocyte ratio (PLR) and C reactive protein (CRP) [7, 11].

Besides PNI, multivariable analysis (Table 3) showed that age, primary tumor site, pT classification, LND and surgical margin were independent predictors for survival in HPSCC patients. Elders were correlated with early distant metastasis in our study (P = 0.003). The survival of patients with advanced pT [14, 16] or primary tumors originating from posterior wall or postcricoid regions [14] are worse. Our results were consistent with these studies. Positive pN in HPSCC is regarded as a poor factor for survival [13, 16]. Due to extensive lymphatic network and submucosal spread, HPSCC frequently presents as lymph node metastasis of neck. There were 83.7% of 123 patients in our study had nodal metastasis in neck, it was similar to 79% of nodal metastasis reported by Zhejiang Cancer Hospital [16]. Previous studies confirm that metastatic LNs > 3 predicts early relapse and distant metastasis [3, 14]. The lymph node density (LND) is calculated as the number of metastatic lymph nodes divided by the total number of lymph nodes removed. It incorporates the burden of nodal disease with the extent of nodal dissection, which was shown to have prognostic value in HPSCC patients [15, 17]. Our study revealed that LND > 0.06 was correlated with early distant metastasis (P = 0.013), tumor progression (P = 0.007) and a trend of worse OS (P = 0.070). The poor impact of positive surgical margin [15] on survival outcomes was demonstrated as independent predictors in HPSCC, we drew same result in OS (P = 0.034) and LRFS (P = 0.017). Nevertheless, the significance of nodal ECS [14, 16, 17], perineural invasion [15] and lymphovascular invasion [14, 15, 18] in prognosis need to be verified with expansion of population.

The strengths of this study were uniform grouping criteria and treatment modalities. Moreover, clinicopathological prognostic factors were included into analysis and compared between PNI groups to exclude confounders. The major limitations were its retrospective nature, relatively small size with all patients enrolled from single institution, and short mean follow up duration. Further large prospective randomized clinical trial in multicenter setting should be conducted to confirm the prognostic impact of preoperative PNI in HPSCC patients with radical treatment.

## Conclusion

Preoperative PNI is an independent prognostic factor in HPSCC patients treated with radical surgery. High preoperative PNI predicts better outcomes. Since PNI can objectively reflect the heterogeneity of individual, it can be used together with the conventional TNM staging system for prognostic prediction and in determining treatment strategies. For patients with low preoperative PNI, nutritional intervention preoperatively and/or more intensified adjuvant therapy should be considered.

## Abbreviations

AJCC: American Joint Committee on Cancer
CI: confidence interval
CRP: C reactive protein
CRT: combined chemoradiotherapy
CT: computed tomography
CTV: clinical target volume
DMFS: distant metastasis-free survival
ECS: extracapsular spread
HNSCC: head and neck squamous cell carcinoma
HPSCC: hypopharyngeal squamous cell carcinoma
HR: hazard ratio
IMRT: intensity-modulated radiotherapy
LN: lymph node
LND: lymph node density
LRFS: locoregional recurrence-free survival
MRI: magnetic resonance imaging
NLR: neutrophil to lymphocyte ratio
OS: overall survival
PFS: progression-free survival
PLR: platelet to lymphocyte ratio
pN: pathological nodal
PNI: prognostic nutritional index
pT: pathological tumor
Ref: reference
RT: radiotherapy
SPECT: single-photon emission computed tomography
TNM: tumor-node-metastasis
